# A study on how concurrent visual feedback affects motor learning of adjustability of grasping force in younger and older adults

**DOI:** 10.1038/s41598-022-14975-4

**Published:** 2022-06-24

**Authors:** Ryohei Yamamoto, Kazunori Akizuki, Kazuto Yamaguchi, Jun Yabuki, Tatsuya Kaneno

**Affiliations:** 1 Department of Rehabilitation, Kyushu University of Nursing and Social Welfare, Kumamoto, Japan; 2 Department of Physical Therapy, Kobe International University, Hyogo, Japan; 3 Department of Physical Therapy, Ibaraki Prefectural University of Health Sciences, Ibaraki, Japan; 4 Department of Physical Therapy, Ibaraki Prefectural University of Health Sciences Hospital, Ibaraki, Japan; 5 Department of Occupational Therapy, Mejiro University, Saitama, Japan

**Keywords:** Human behaviour, Geriatrics, Rehabilitation

## Abstract

In this study, we investigated the differences of the effectiveness from concurrent visual feedback among younger and older adults in learning tasks that require adjustability of grasping force (AGF), as well as the functions related to AGF in each generation. The younger and older adult groups were evaluated for simple visual reaction time as visual-motor speed (VMS) and a 100 g AGF task that reflected the difference between desired performance and actual performance. The main learning task was then practiced using concurrent visual feedback and tested without feedback. The VMS of older adults was slower than that of the younger, and the error in the 100 g AGF task was larger in older adults than in the younger adults. Performance improved from pre-test to retention test in both groups, but the older adult group failed to reach the level of the younger adult group. The results of this study show that concurrent visual feedback is effective for learning the tasks that require AGF in both groups. Indicatively, improvement in performance during practice is insufficient in older people for whom there is a large difference between desired performance and actual performance, or whose VMS is slow.

## Introduction

In activities of daily living, it is necessary to grasp the object with an appropriate force according to the shape and weight of the object, and fine adjustment of the force is required. It is known that older adults have less ability to maintain stable force, and that upper limb function declines with ageing^1^. Previous studies have defined this ability as the adjustability of grasping force (AGF) and reported that older people showed lower AGF than younger people^2,3^. It has been reported that dexterity of the upper limbs is associated with activities of daily living (ADL) using evaluations of upper limb function^4,5^. Furthermore, it has been reported that in older adults, as with items related to upper limb function in ADL, there are restrictions on handling of small objects such as coins and buttons, as well as finger movements such as answering telephone calls and meal preparation^6^. In addition, Rodríguez-Aranda et al.^7^ reported that there was no difference between the movement of the upper limbs of older adults and that of young adults as a kinematic feature during peg operation, but there was a difference in the movement of fingers such as gripping a peg. From these facts, it is considered that the ability to grasp with appropriate force is impaired with ageing, and it is more likely that ADL, which are strongly related to the functions of fingers, cannot be performed by oneself. By establishing a practice method that promotes acquisition of tasks that require AGF in older adults, improvement in the degree of independence in the daily life of the older adult is expected.

Motor learning is the process of learning some kind of movement from experience, such as practice and holding it for a long period of time. Raz et al.^8^ reported that older people had lower motor performance than younger people when using a one-handed position adjustment task called the pursuit rotor task, which tracks a metal stylus against a moving target. It has also been reported that older adults are less likely to develop motor learning than young adults^9,10^, and it takes them more time to acquire skills than young adults^11–13^. Thus, it is claimed that motor learning itself is possible in older adults, although it takes longer than in the young^14–17^. However, motor learning in older adults has been shown to be more interindividual than in young adults^13,18^. This may be because various functional declines appear with ageing in older adults, and the degree of functional deterioration varies from person to person. First, when grasping an object in a new environment, it is necessary to adapt the condition of the fingers to that environment, but it has been reported that there is no significant difference between young and older people in grasping adaptation^19^. Next, memory is said to be associated with motor learning among the functions that decline with age, but the effects of general memory impairment are known to be small^20–22^. Schaefer et al.^23^ reported that visuospatial impairment was associated with learning the functional reach task, which includes manipulating objects with the non-dominant hand, in older adults. In other words, it is expected that cognitive function directly related to the task is influencing for degree of learning.

A study on AGF reported that the AGF of older adults was lower than that of younger adults using a method of approximating the grasping force to the target value displayed on the screen^1^. This method of giving visual information in parallel with movement during practice is called concurrent visual feedback. Visual feedback is one of the methods used to generate motor learning, and is employed in various fields such as rehabilitation and sports^24–27^. Effectively obtaining information related to a task is important for learning new movements^28^. Concurrent feedback given in parallel with movement improves performance during practice in simple tasks, but degrades motor learning^29–31^. Contrarily, a comparison of cognitive load aspects reveals that concurrent feedback has less cognitive load than terminal feedback^32^ and has been reported to be effective for learning complex tasks that tend to increase cognitive load^33,34^. However, Krishnan et al.^35^ reported that older people had less effectiveness for motor learning from concurrent feedback practice than younger people in learning new gait patterns. From these facts, cognitive load during the task is likely to be reduced by practicing in an AGF measurement environment using concurrent feedback, and motor learning will occur even in older adults, but the degree will be lower than that in younger adults. However, the differences between generations in motor learning of AGF tasks have not been examined.

Next, as mentioned above, measurement of AGF is the same as practicing using concurrent feedback, and the performance is expected to be influenced by the speed at which visual information is reflected in movement and the ability to properly adjust the grasping force without relying on the external information. The speed at which visual information is reflected in movement is called visual-motor speed (VMS) and is measured using a simple visual reaction time (VRT) in a computer-based neurobehavioural evaluation system^36^. Many studies have reported that simple VRT increases with age^37–39^. In addition, the function of appropriately adjusting competence without relying on external information is considered to be the difference between the desired and actual performance. Reportedly, ageing causes a decrease in functions such as reaction speed and accuracy of movements, and the degree of this difference is likely to increase^40^. However, association of these functional declines with the performance of AGF tasks using visual information in older adults has not been investigated. It has not been examined whether practice using concurrent visual feedback, which is effective for motor learning in older adults using other learning tasks, is effective for learning AGF tasks. In addition, the effectiveness of practice with concurrent visual feedback, which has been reported to be effective for motor learning in older adults, has not been examined for learning tasks that require AGF.

In this study, we investigated the effect of practice using concurrent visual feedback on learning AGF tasks in older people. Furthermore, we investigated how the VMS and degree of difference between the desired and actual performance were related to the AGF. Our first hypothesis is that older adults will be slower at learning AGF tasks under concurrent visual feedback compared to younger adults. The second hypothesis is that the performance of AGF tasks in older adults during practice with concurrent visual feedback is affected by VMS and the degree of difference between the desired and actual performance.

## Method

### Participants

The eligibility criteria for the young participants included in the study were 20 ≤ age < 30 years, and were affiliated with the Faculty of Nursing and Welfare, the Kyushu University of Nursing and Social Welfare, Japan. The exclusion criteria included having an experience of carrying out tasks similar to those required to be performed for this study, or having a current or past (history of) orthopaedic or neurological disease of the hands and fingers that impacted ADL. The eligibility criteria for the older individuals were age ≥ 60 years, living in their own homes (main place of residence), able to ambulate without a walking aid, and able to travel to the study venue by themselves. The exclusion criteria for the latter group were having an experience of carrying out tasks similar to those required for this study, having a current or past (history of) an orthopaedic or neurological disease of the hands and fingers that impacted ADL, or scoring ≤ 23 on the Mini-Mental State Examination.

A power analysis was conducted to estimate the sample size using G*Power 3.1.9.7. The sample size calculation was considered a power calculation to detect differences between the groups in the performance of a task that required AGF during the test sessions. We used repeated measured analysis of variance (ANOVA), within-between interaction with an α error level probability of 0.05, and a power (1‐β error probability) of 80%. The medium effect size Cohen was set to f = 0.25. The analysis described above revealed that a total sample size of 34 was required for this study. Therefore, recruitment was closed when 17 applicants from each group were confirmed. As a result, 36 healthy individuals (28 men and 8 women) participated in this study. The participants were divided into two age groups. The young adult group (14 women, 4 men) were aged between 20 and 22 years (mean age = 21, SD = 0.3), and the older adult group (14 women, 4 men) were aged between 60 and 75 years (mean age = 71, SD = 3.9).

None of the participants had reported any neurological or vestibular disorders or orthopaedic conditions before participating in the study. The participants had no prior experience with the learning task, and they were not informed of the specific purpose of our study. A preliminary explanation of the study details was provided to all participants, and written informed consent was obtained. Our study protocol was approved by the Institutional Review Board of Kobe International University (G2019-102) and performed in accordance with the Declaration of Helsinki.

### Equipment

**Figure 1:**
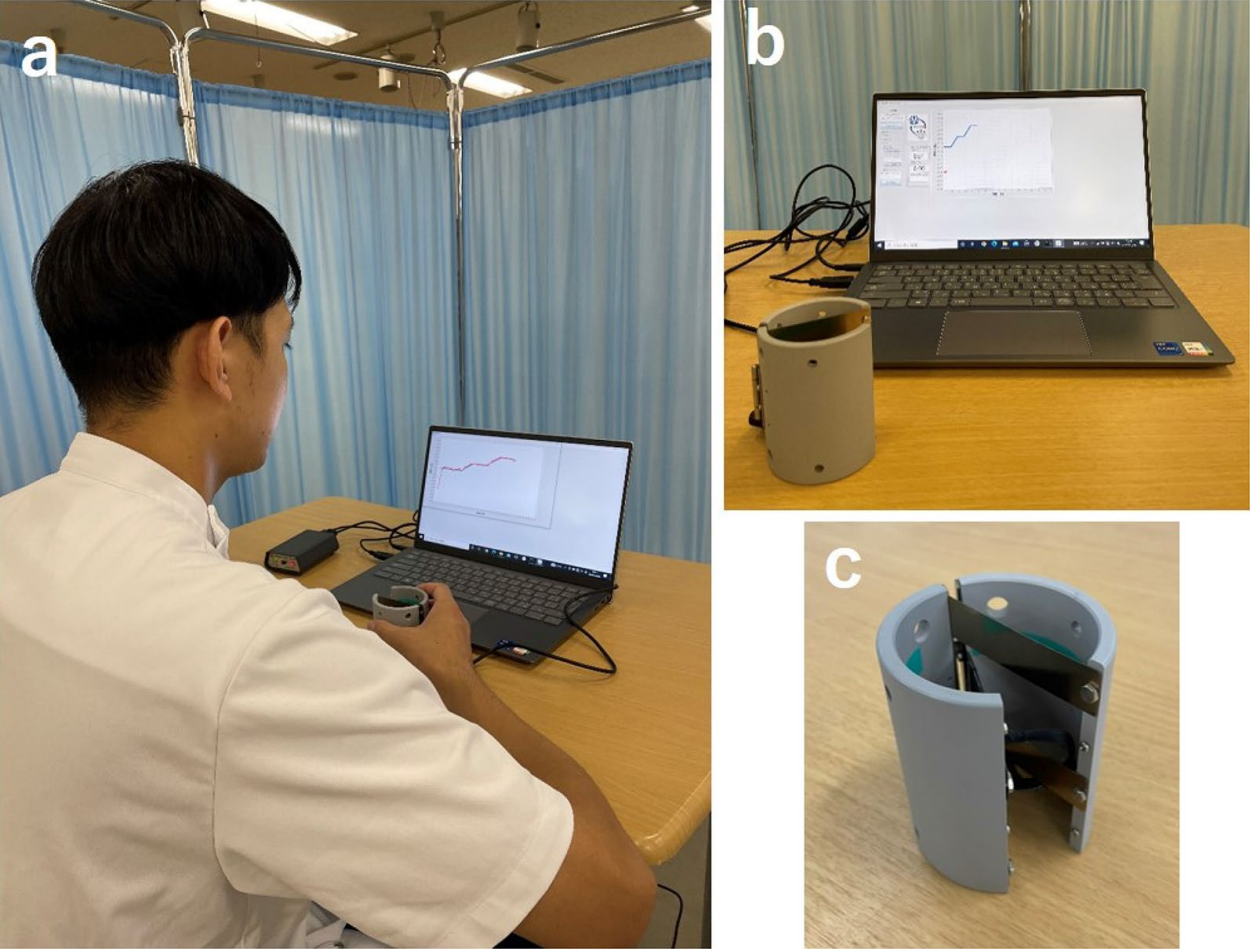
iWakka. (**a**) State when using iWakka, (**b**) iWakka and monitor/computer displaying feedback, (**c**) iWakka.

In this study, iWakka (Aimu Co., Ltd.) was used to measure AGF based on a report by Kaneno^2,3^ (Fig. 1). Written informed consent for publication of the image in Fig. 1 was obtained from one of the authors. iWakka is a cylindrical device with a height of 80 mm and a diameter of 65 mm and can measure a grasping force of 0–400 g depending on the degree of opening and closing of the device. During measurement, each participant held the iWakka with one hand whilst in a sitting position and continuously adjusted the grasping force to approximate an arbitrarily set target value. The measured value and the target value during measurement can be displayed as feedback on a monitor placed in front of the participant. Since the AGF is calculated as the root mean square error (RMSE) from the absolute error per unit time between the measured value and the target value, a smaller absolute value is indicative of a better AGF.

### Procedure and tasks (VMS, 100 g AGF task and main learning task)

**Figure 2:**
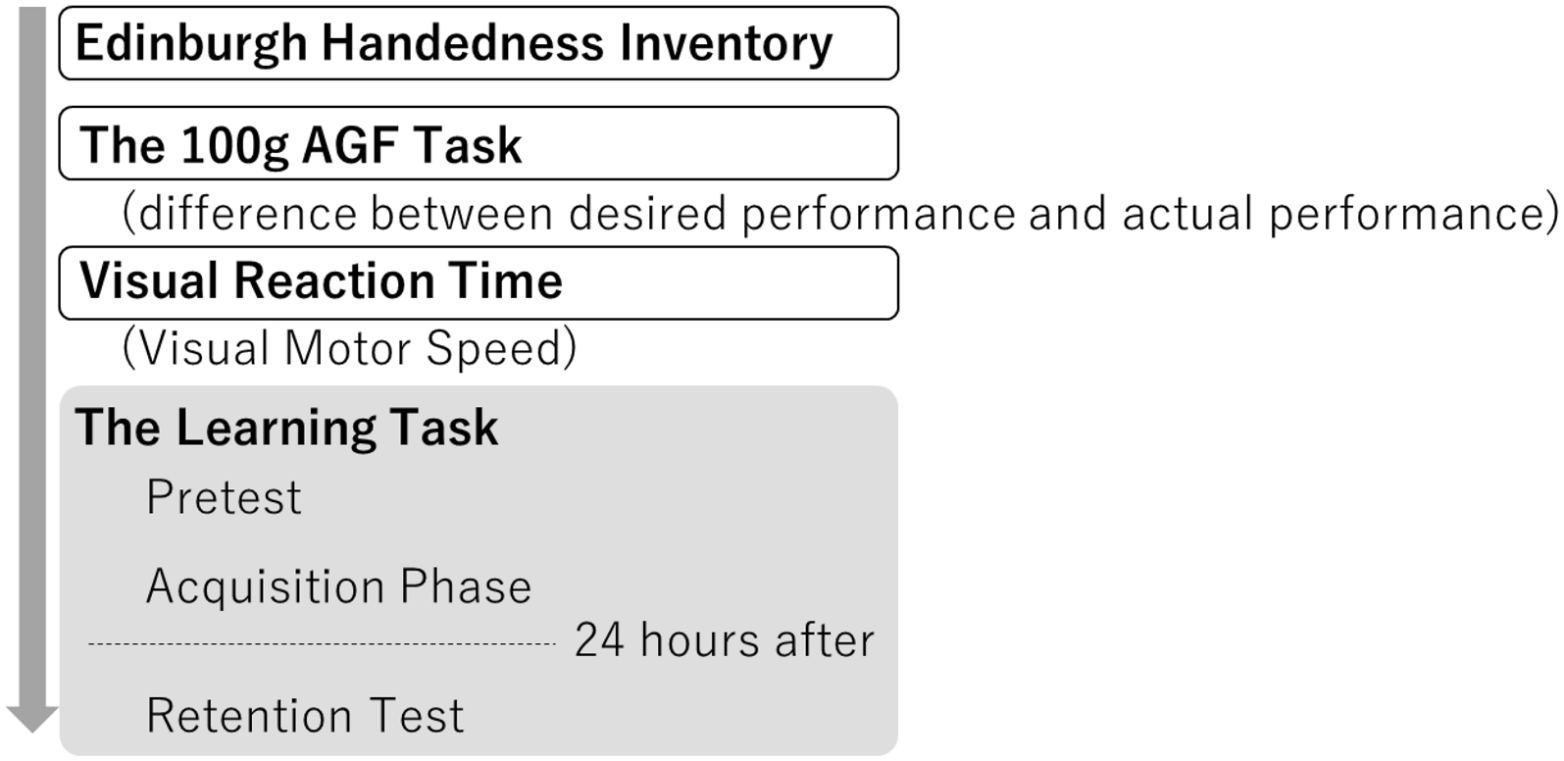
Procedure. In the 100 g AGF task, 5 trials were performed without giving feedback. The number of VRT measurements was 5 trials. Regarding the main learning task, the pre-test was conducted in 4 trials without feedback. The acquisition phase consisted of 4 trials × 3 blocks for a total of 12 trials, and concurrent feedback was given during the performance. The retention test was conducted about 24 h after the acquisition phase in the same manner as the pre-test.

The older and young adult groups were measured for 2 days using the same procedure (Fig. 2). Prior to the measurement, the dominant hand of each participant was determined using the Edinburgh Handedness Scale. In this study, the learners performed all the tasks with their non-dominant hands. As a result of the Edinburgh Handedness Scale, all the participants were right-handed, so they performed the task with their left hand.

After determining the dominant hand, the 100 g AGF task was measured. Two AGF tasks were used in the study. First, a 100 g AGF task was set as an index of difference between the desired and actual performance in the state, excluding the visual information and the temporal aspect. In this task, the participant was asked to adjust the measurement value of iWakka to 100 g for 10 s without the feedback displayed on the monitor. No feedback was given to prevent the participant from correcting the difference between the desired performance and actual performance.

After the completion of the 100 g AGF task, the visual simple reaction time was measured. In this study, referring to the report by Cuthbertson et al.^41^, VRT was measured as an index that reflected VMS using the website www.humanbenchmark.com. We performed this task using a computer that could connect to the Internet. The participants were asked to click the mouse as soon as possible when the colour of the front screen automatically changed from red to green. The time (milliseconds) from when the screen turned green to when the participant clicked the mouse was automatically calculated. In this study, VRT was measured five times.

**Figure 3:**
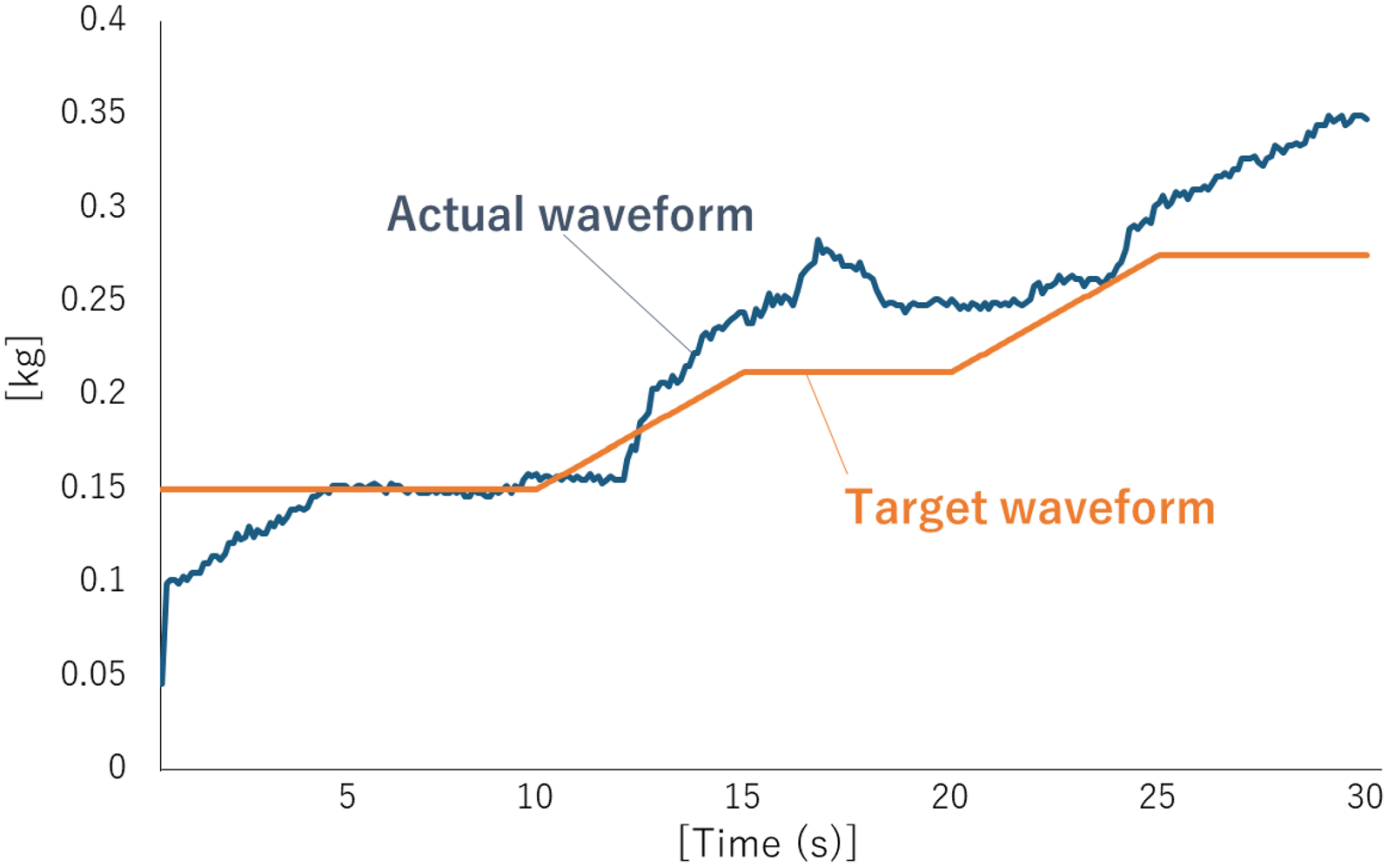
Waveforms of the main learning task and feedback screen. This is the waveform of an individual participant in the young adult group.

Next, tests and practice were conducted on the main learning tasks. In this task, as shown in Fig. 3, participants were asked to continuously adjust their gripping force to match the target value without relying on visual information. The target value was changed in a stepwise manner at certain time intervals. The displayed values moved from right to left on the graph with the passage of time. The task consisted of 30 s per trial. The procedure of the main learning task consisted of a pre-test, an acquisition phase, and a retention test. In the pre-test, the participants performed four trials of the task without feedback. The acquisition phase consisted of three blocks, with 4 trials in one block. The participants performed a total of 12 trials in the acquisition phase. During the acquisition phase, the participant was given concurrent feedback by confirming the target value and the actual measured value displayed on the monitor. A 20-s break was set between each trial, and a 60-s break was set between each block. The retention test was conducted 24 h after completion of the acquisition phase with identical contents to the pre-test.

### Outcome measures and statistical analysis

In this study, the RMSE of the 100 g AGF task and the main learning task were used as parameters of the AGF. For the 100 g AGF task, the RMSE was calculated from the target value and the measured value, with 5–10 s of the measurement time as the analysis range, and the average value of five trials was calculated. Next, in the main learning task, the RMSE was calculated with a measurement time of 5–30 s as the analysis range, and the average value was calculated in the pre-test, each block of the acquisition phase, and the retention test. In addition, VMS was calculated as the average value of the five trials completed by each participant.

Statistical analyses were conducted using IBM SPSS Statistics 25 (IBM Corp., NY, USA) for Windows. The RMSE of the pre-test and retention test were analysed using a 2 (generation: younger adult versus older adult) × 2 (test: pre-test versus retention test) ANOVA with repeated measures on the last factor. When a significant main effect and interaction was obtained, the paired sample t-test and the independent sample t-test were performed as post hoc tests. Independent sample t-tests were also performed on the 100 g AGF task and VMS in the older adult and younger adult groups.

In addition, a multivariable linear regression analysis was performed to test the hypothesis that the performance of older adults in AGF tasks during practice with concurrent visual feedback is affected by VMS and the degree of difference between the desired and actual performance. In this analysis, a stepwise method was used, with the RMSE of each block in the acquisition phase as the objective variable and the 100 g AGF task and VMS as the explanatory variables. Furthermore, this analysis was also performed on the data collected from younger adults to confirm whether the same events were observed in them. Statistical significance was set at p < 0.05.

### Ethics declarations

We have no ethical concerns to declare.

## Results

### RMSE of the main learning task in the pre-test and retention test

**Figure 4:**
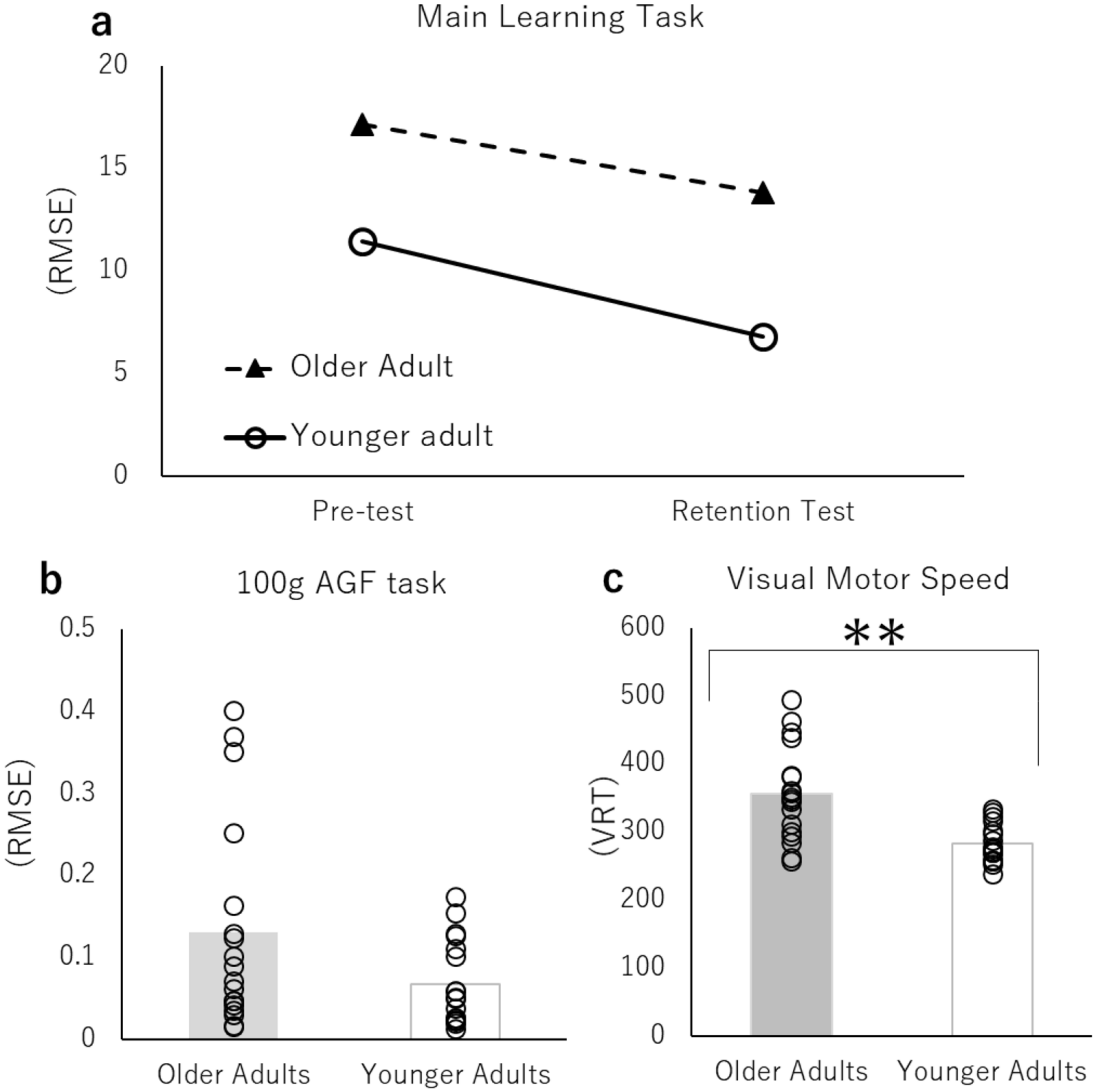
Results of each measurement item. (**a**) RMSE of the pre-test and the retention test (the main learning task), (**b**) Comparison of the 100 g AGF task, (**c**) comparison of VMS, (*p < 0.05, **p < 0.01).

A two-way ANOVA of 2 (generation: younger adult versus older adult) × 2 (test: pre-test versus retention test) was performed, with the RMSE of each test as the objective variable for the older and younger adult groups (Fig. 4a). No significant interaction was observed. A significant main effect was found for the factors within the participant (F (1, 34) = 7.48, p < 0.05, ηp^2^ = 0.180). These results indicated that the younger adult group showed significantly lower values than the older adult group in both the pre-test and the retention test. In addition, a significant main effect was observed for the factors between participants (F (1, 34) = 5.73, p < 0.05, ηp^2^ = 0.144). These results further revealed that both the younger and the older groups showed significantly smaller values in the retention test than in the pre-test.

### The 100 g AGF task

Independent sample t-tests were performed on the older and younger adult groups, with the RMSE of the 100 g AGF task as the objective variable (Fig. 4b). No significant difference was observed between the groups. However, a moderate effect size was observed (p = 0.061, difference in mean 0.06, 95% CI [− 0.00313, 0.13], Cohen's d = 0.66).

### Visual-motor speed

Independent sample t-tests were performed on the older and younger adult groups using VRT as the objective variable (Fig. 4c). As a result, the younger adult group showed significantly smaller values than the older adult group (p < 0.001, difference in mean 73.56, 95% CI [37.28644, 109.82], Cohen's d = 1.51).

### The multivariable linear regression analysis of RMSE of the acquisition phase

Multivariable linear regression analysis was performed, with RMSE as the objective variable and the 100 g AGF task and VRT as explanatory variables in each block of the acquisition phase (Table 1). As a result, the following model was calculated for each block in the older adult group: First, in block 1, a model called RMSE = 2.598 + 18.571 × 100 g AGF task was calculated (R = 0.639, R^2^ = 0.408, p < 0.01). In block 2, the model of RMSE = − 0.195 + 0.003 × VRT was calculated (R = 0.495, R^2^ = 0.245, p < 0.05). Finally, in block 3, the model RMSE = − 0.709 + 0.004 × VRT was calculated (R = 0.503, R^2^ = 0.253, p < 0.05). In the younger adult group, no significant model was calculated for any of the blocks.

**Table 1:** The multivariable linear regression analysis of each block in the acquisition phase for the older adult group.

RMSE of each block	SE β (95% CI)	B	p-value
**Block1**			
100 g AGF task	0.639(6.711–30.432)	18.571	0.004
VMS	–	–	0.442
R^2^	0.408		
F	11.018		
**Block2**			
100 g AGF task	–	–	0.240
VMS	0.495(0.000–0.005)	0.003	0.037
R^2^	0.245		
F	5.195		
**Block3**			
100 g AGF task	–	–	0.631
VMS	0.503(0.000–0.007)	0.004	0.033
R^2^	0.253		
F	5.419		

*SE β* standardised coefficient, *CI* confidence interval, *RMSE* Root Mean Square Error, *VMS* visual-motor speed.

## Discussion

Older adults have been reported to perform worse than younger people in AGF tasks using visual information^2,3^. However, the effect of the ability to properly adjust the grasping force without relying on external information and the speed to reflect visual information in movement has not been investigated. In addition, differences between older and younger adults in the effect of practice in an AGF measurement environment on motor learning of AGF tasks has not been investigated. Therefore, we investigated the differences between younger and older adults in the effect of practice using concurrent visual feedback on learning AGF tasks, as well as how VMS and the difference between the desired and actual performance affected AGF in older adults.

As a result of this study, the older adult group effectively enhanced motor learning of the AGF task by practising with concurrent visual feedback, as in the younger adult group. Smith et al.^42^ reported that procedural learning was not significantly impaired in older adults. A previous study reported that concurrent visual feedback was effective for motor learning in the older adult group in a coordinated task on both upper limbs. In other words, it showed the possibility of motor learning in older adults and the effect of practice with concurrent visual feedback. However, although motor learning occurred in both groups, the older adult group could not reach the level of the younger adult group. Certain reports establish that it is difficult for the older adult to learn the same performance as young people^15,43^. Coats et al.^44^ reported that the learning effects of practising with concurrent visual feedback on the visual coordination task with the upper limb in older adults were about half of that of younger people; this had resulted from age-related decline in visual motion perception. In this study, young people performed better on both VRT and 100 g AGF tasks than older adults. Thus, the results of this study show that concurrent visual feedback is effective for motor learning in older adults, and it is difficult for the older adult to achieve the same performance as the younger adult in the AGF task.

No significant difference was observed in the comparison of the results of the 100 g AGF task between the groups, but the effect size was moderate. It is thus possible that the RMSE of the 100 g AGF task was larger in the older adult group than in the younger adult group. There is also a possibility that the difference between the desired performance and actual performance in older adults is larger when compared with that of the younger adult group. Vieluf et al.^45^ reported that the error in isometric force adjustment when a pinch motion was performed without visually presenting a target value was larger in older adults than in the younger adults. This result is consistent with the results of the 100 g AGF task in this study. Moreover, the older adult group showed a larger VRT value than the younger adult group. This result is consistent with those of many previous studies^37–39^. The results of this study supported previous studies that the sensory response and control of motor output was degraded by the effects of aging.

Regression analysis of the performance of the main learning task during the acquisition phase revealed that the performance of the older adult group was influenced by the performance of the 100 g AGF task in the early stage of the practice, and that of VRT in the latter stage. Galganski et al.^46^ have reported that the variation of motor output in a hand muscle in older adults increased during smaller force control. Furthermore, Welford et al.^47^ stated that ageing increased the level of noise in the motor system, which is a major cause of poor performance. It is possible that older adults, who had a larger difference between the desired performance and actual performance, were influenced the noise of the motor system which lowered their performance at the early stages of practice. Next, regarding VMS and performance during practice, it has been confirmed that the larger the VRT, the lower the performance in the latter stage of practice in older adults. Previous studies have reported that older adults are more likely to generate motion noise when a minor force adjustment is required during force adjustment based on visual information^48^. It can be seen that the task using iWakka required fine adjustment of the force of 400 g or less using visual information, and this tendency was more remarkable. VRT is also used to evaluate premotor time. Premotor time is the time interval measured from the presentation of a stimulus to the onset of muscle activity^49^. This interval consists of several processing phases, including stimulus identification, stimulus processing, response selection, motor plan generation, and motor execution to activate the motor neuron pool. It has been reported that the increase in premotor time in older adults is due to an extended motor plan generation phase^50,51^. In line with the results of previous studies, it is possible that the results of the older adult group in our study, where slower VMS was attained, were due to delayed motor plan generation based on visual feedback information. From these facts, it takes time for the older adult to correct the difference between desired performance and actual performance at the initial stage of the practice. Furthermore, the degree of improvement by the practice is small in older adults with a slow VMS. In the younger adult group, no significant regression equation was obtained with the same variables.

This study has some limitations. First, the sample size for this study was small. A moderate effect size was adopted for this study as no reference study was found in the sample size calculation. If the results of any study are based on an inadequate sample size, the statistical analysis results may be overestimated. This means it may be difficult to determine if the study’s outcome is true or if the study’s findings may be extrapolated to the larger population. Thus, we suggest that further studies with larger sample sizes be conducted to clarify the relationship between the various functions that we submit decrease with aging and motor learning for AGF. Second, in this study, it was clarified that the VMS and the difference between desired and actual performance had effects on the performance during the practice, but these functions were not measured the day after that. It is necessary to confirm whether these functions are changed by practice with concurrent visual feedback and investigate whether this degree is related to the motor learning effect. Third, VRT was used as an index of VMS to eliminate motor elements such as shoulder joints. However, the Repeatable Battery for the Assessment of Neuropsychological Status (RBANS) has been reported to be related to motor learning for older adults^52^. The RBANS assesses cognitive function and contains five subtests of immediate and delayed memory, visuospatial/constructional, attention, and language. Among them, visuospatial functioning is related to motor learning in older adults. The Purdue Pegboard Test (PPT) is an assessment tool that measures the coordination between upper limb movement and visual information. The PPT has been reported to be sensitive to aging and may be considered a measurable motor index of dexterity even in older adults with high cognitive function^53^. It has also been reported that audio-visual integrative training has the potential to improve the motor and cognitive functioning of older adults^54^ and the results of the PPT could be improved by practicing motor tasks intended for the upper limbs. In the future, by using these indicators, it will be possible to examine the relationship between AGF and mental function, which cannot be achieved by VRT.

Finally, Kaneno et al.^2^ reported that the older adult had lower AGF than younger individuals in situations in which concurrent visual feedback was used, as in the acquisition phase of this study. The authors state the need for an intervention regarding this ability. The results of this study showed that the difference between desired performance and actual performance, and VMS was associated with a decrease in AGF. Therefore, intervention for these functions before the start of the practice of the learning task may lead to improvement of AGF and promotion of the motor learning effect.

## Conclusion

In this study, we investigated whether the difference between desired and actual performance and VMS was related to AGF in older adults and investigated the effect of practice using concurrent visual feedback on AGF tasks. As a result of this study, it was clarified that although older adults could learn the AGF task by practicing with concurrent visual feedback, the performance did not reach the level of the younger adults. It was also found that in practice using concurrent visual feedback, the performance in the early stages was affected by the difference between desired performance and the actual performance, and by VMS as the practice progressed. The results of this study provide new insights that facilitate the learning of AGF tasks in older adults.

## Data Availability

Data is available upon reasonable request to the corresponding author.
